# *gdf6a* Is Required for Cone Photoreceptor Subtype Differentiation and for the Actions of *tbx2b* in Determining Rod Versus Cone Photoreceptor Fate

**DOI:** 10.1371/journal.pone.0092991

**Published:** 2014-03-28

**Authors:** Michèle G. DuVal, A. Phillip Oel, W. Ted Allison

**Affiliations:** 1 Department of Biological Sciences, University of Alberta, Edmonton, Alberta, Canada; 2 Centre for Prions and Protein Folding Disease, University of Alberta, Edmonton, Alberta, Canada; 3 Department of Medical Genetics, University of Alberta, Edmonton, Alberta, Canada; National Institutes of Health/NICHD, United States of America

## Abstract

Functional vision restoration is within reach via stem cell therapy, but one of the largest obstacles is the derivation of colour-sensitive cone photoreceptors that are required for high-acuity daytime vision. To enhance progress made using nocturnal murine models, we instead utilize cone-rich zebrafish and herein investigate relationships between *gdf6a* and *tbx2b* in cone photoreceptor development. Growth/differentiation factor 6a (*gdf6a*), a bone morphogenetic protein family ligand, is an emerging factor in photoreceptor degenerative diseases. The T-box transcription factor *tbx2b* is required to specify UV cone photoreceptor fate instead of rod photoreceptor fate. Interactions between these factors in cone development would be unanticipated, considering the discrete phenotypes in their respective mutants. However, *gdf6a* positively modulates the abundance of *tbx2b* transcript during early eye morphogenesis, and we extended this conclusion to later stages of retinal development comprising the times when photoreceptors differentiate. Despite this, *gdf6a^−/−^* larvae possess a normal relative number of UV cones and instead present with a low abundance of blue cone photoreceptors, approximately half that of siblings (p<0.001), supporting a differential role for *gdf6a* amongst the spectral subtypes of cone photoreceptors. Further, *gdf6a^−/−^* larvae from breeding of compound heterozygous *gdf6a^+/−^*;*tbx2b^+/−^* mutants exhibit the recessive *lots-of-rods* phenotype (which also shows a paucity of UV cones) at significantly elevated rates (44% or 48% for each of two *tbx2b* alleles, χ^2^ p≤0.007 for each compared to expected Mendelian 25%). Thus the *gdf6a^−/−^* background sensitizes fish such that the recessive *lots-of-rods* phenotype can appear in heterozygous *tbx2b^+/−^* fish. Overall, this work establishes a novel link between *tbx2b* and *gdf6a* in determining photoreceptor fates, defining the nexus of an intricate pathway influencing the abundance of cone spectral subtypes and specifying rod vs. cone photoreceptors. Understanding this interaction is a necessary step in the refinement of stem cell-based restoration of daytime vision in humans.

## Introduction

The genetic regulation of cone photoreceptor differentiation from retinal progenitor cells is a critical knowledge gap hindering stem cell therapy as a feasible solution for clinical vision restoration. Such therapies promise treatment in patients with a breadth of retinal disease including retinitis pigmentosa and macular degeneration. Identifying pathways that promote cone photoreceptor fates, rather than rod photoreceptor fates, is particularly critical due to the reliance of the human visual system on cones for its most important functions: daytime vision, colour discrimination and high visual acuity.

Apart from this, current efforts to refine stem cell therapy more prominently include the identification of intrinsic genetic factors that regulate progenitor fate. Sorting of photoreceptor progenitor cells for implantation is the most efficient contemporary approach, employing expression of photoreceptor lineage-specific genes (e.g. *NRL*, *CRX*, and *NEUROD*) to facilitate the isolation of progenitor cells destined to develop into the photoreceptors of interest [1]–[6]. From this perspective, the current list of genes with roles in cone development remains too short for the purpose of development of functional cones that can integrate into an existing retinal structure, thereby sufficiently restoring functional daytime vision. This list consists largely of *TRβ*, *RXRγ*, *RORα*, *RORβ*, *COUP-TF*, [4], [7]–[10] and *tbx2b* [11].

The functions of photoreceptor genes have largely been investigated in mice, especially in the context of degenerative disease [6], [12]–[14], however the innately low cone photoreceptor density in murine models has meant that an understanding of cone photoreceptor specification has lagged behind that of rod photoreceptors. A complementary animal model promises to expand the list of genes and regulatory pathways in cone photoreceptor development: the zebrafish. The retina of zebrafish is structurally and functionally conserved to that of humans, and, due to the diurnal nature of zebrafish, it is cone-rich akin to the human macula. Zebrafish possess rods and four cone spectral subtypes (ultraviolet- (UV-), blue-, green- and red-sensitive cones), which are spatially arranged in a highly regular heterotypical mosaic [15]–[21]. In addition, zebrafish undergo external development, allowing for ease of observation and experimental manipulation, supported by a diverse genetic toolbox (e.g. mutants and transgenics). Of particular benefit to the study of stem cell therapy is the robust intrinsic regenerative capacity of the zebrafish CNS, which is the target of enthusiastic scrutiny [22]–[32]. In further pursuit of understanding this regenerative capacity, we have recently engineered conditional ablation of cone photoreceptors and argue that spatial cues of the remaining photoreceptor cells have substantial influence on the identity of regenerating photoreceptors [33].

Considering the great promise of zebrafish to become the premier model of photoreceptor regeneration, it is surprising that few regulatory factors in photoreceptor development are yet to be characterized in fish. One example is thyroid hormone, which initiates UV cone death and regeneration in trout [34]–[37] and modulates the maximal wavelength sensitivity of cones in zebrafish [16]. Building on this, thyroid hormone receptor β has been shown to effect cone specification in mice [14], [38] and more recently in zebrafish [39]. We proposed that, during trout cone photoreceptor regeneration, thyroid hormone modulates a switch in progenitor specification very much akin to that described below, wherein UV cones are produced at the expense of rod photoreceptors [34].

Another regulator of photoreceptor fate described in zebrafish is *tbx2b*, a transcription factor of the T-box family homologous to the mammalian gene *TBX2*. *Tbx2b* is required for neuronal differentiation in early retinal development and for maintaining dorsal retina identity during patterning of the dorsal-ventral axis [40]. Mutation of *Tbx2* in mice results in microphthalmia [41]. This is in agreement with its position downstream of bone morphogenetic protein 4 (*BMP4*), mutations in which cause microphthalmia in humans and mice. Of great interest herein, *tbx2b* plays a role in promoting UV cone fate vs. rod fate late in zebrafish retinal development, as demonstrated by excess rods and few UV cones (denoted as the “*lots-of-rods*” phenotype) in *tbx2b* mutant fish [11]. One recessive allele, *tbx2b^fby^* (also known as *tbx2b^c144^*), is reasonably considered to be a null allele due to a nonsense mutation in the sequence encoding its DNA-binding T-box domain [42]. Homozygous mutants of *tbx2b^fby^* exhibit a severe form of the *lots-of-rods* phenotype, wherein few or no UV cones can be detected. A second recessive allele is *tbx2b^lor^* (also known as *tbx2b^p25bbtl^*), presumed to be a hypomorph because it generates a less severe form of the *lots-of-rods* phenotype, exhibiting a substantial reduction in the abundance of UV cones compared to wild type fish, but not to the degree observed in *tbx2b^fby^* mutants. The location and nature of the *tbx2b^lor^* mutation is unknown; however, based on linkage analysis and its failure to complement the *tbx2b^fby^* allele, it is inferred to be near the coding region for *tbx2b*, but not within it (see [11] and Results herein).

In recent studies, we and others identified *gdf6a* as a candidate regulator of cone photoreceptor development and disease [43]–[45]. *Gdf6a* is a BMP gene in the transforming growth factor β (TGFβ) ligand super-family; *gdf6a* induces dorsal retina fate during ocular morphogenesis, lying upstream of other dorsal patterning genes. Disruption of human *GDF6* and homologs in mice, Xenopus or zebrafish produces anophthalmia, microphthalmia and coloboma with varying degrees of penetrance and severity [43], [45]–[49]. The recessive *gdf6a* null allele used in this study, *gdf6a^s327^*, causes microphthalmia in homozygous zebrafish mutants. Zebrafish knock-downs and mutants of *gdf6a* have down-regulation of *tbx2b* early in retinal development [45], [47], [50], while over-expression of *gdf6a* likewise increases expression of *tbx2b* in the developing zebrafish retina [45], indicating a tight regulation of *tbx2b* transcription by *gdf6a*. Based on this evidence, *gdf6a* is upstream of *tbx2b* in a pathway of dorsal retina patterning; however zebrafish mutants of *tbx2b* (*tbx2b^lor/lor^* and *tbx2b^fby/fby^*) do not exhibit microphthalmia.

Mutations in *GDF6* were recently found to be associated with age-related macular degeneration and Leber's congenital amaurosis, both representing photoreceptor degenerative disease [43], [44]. Further, we demonstrated that the retinas of zebrafish *gdf6a^s327/s327^* mutants exhibit photoreceptor deficits [43], together indicating that disruption of *GDF6* leads to photoreceptor degeneration, which marks *gdf6a* as a potential regulatory factor in the differentiation and/or maintenance of cone photoreceptors.

These commonalities between *gdf6a* and *tbx2b*, including both in early ocular morphogenesis and photoreceptor differentiation/maintenance, led us to hypothesize that *gdf6a* may also modulate *tbx2b* during the regulation of UV cone and/or rod photoreceptor fate specification. Establishing this type of genetic pathway in photoreceptor development would impact the direction of future studies by offering a much-needed springboard toward uncovering further signaling pathways and genetic interactions specific to cone photoreceptors. With such knowledge, stem cell therapy can be refined to procure more cone photoreceptors than using current methods, thereby enhancing functional, daytime vision restoration.

In this study we examined the relationship between the roles of *gdf6a* and *tbx2b* in photoreceptor development. We determined that these two genes do not share a genetic interaction in microphthalmia. Further, while disruption of *gdf6a* does not in itself lead to the predicted disruption of UV cone and rod abundances, *gdf6a* loss-of-function reduces the threshold for *tbx2b* mutations to manifest photoreceptor phenotypes.

## Results

### *Gdf6a* and *tbx2b* do not genetically interact in any apparent way regarding the microphthalmic phenotype

Loss of function in homologues of *GDF6* induces microphthalmia in zebrafish, mice and humans [46], [55]–[58]. Further, mutation of *Tbx2* in mice likewise causes microphthalmia [41]. Considering that *gdf6a* is upstream of *tbx2b* during early eye morphogenesis in zebrafish [45], we hypothesized that simultaneous disruption of both these genes would interact to increase the rate or severity of microphthalmia. We had anticipated that establishing the nature of the genetic interaction between *gdf6a* and *tbx2b* in early eye morphogenesis might be important to provide direction to, or potentially confound, our investigations of *gdf6a* and *tbx2b* in cone photoreceptor differentiation, which occurs later in retinal development.

**Figure 1:**
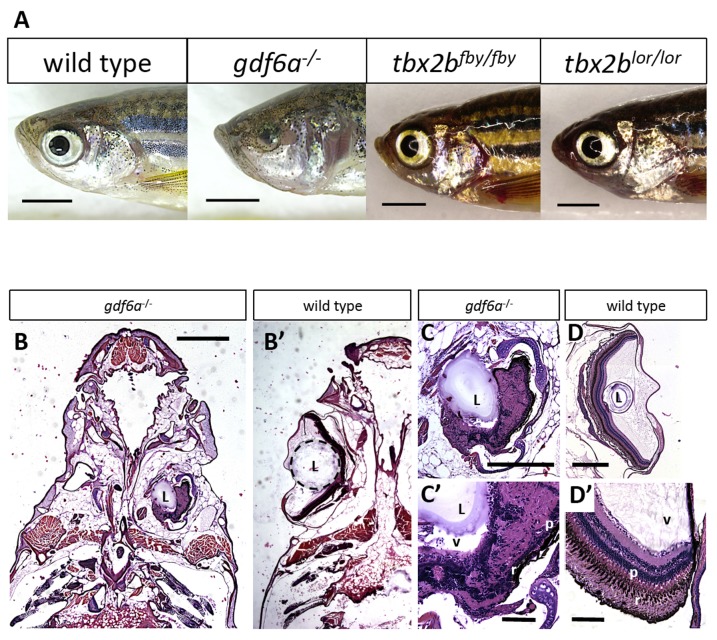
*gdf6a* and *tbx2b* mutants do not share the microphthalmic phenotype, despite a shared pathway in early eye development. A. *gdf6a^s327/s327^* mutants (labeled *gdf6a^−/−^* in figures) exhibit microphthalmia to varying degrees of severity during development and throughout adulthood, unlike their wild type and heterozygous siblings. *tbx2b* mutants do not exhibit microphthalmia, and their eyes develop normally. Scale bars 2 mm. B, C, D. Coronal sections of adult zebrafish heads, comparing microphthalmic *gdf6a^s327/s327^* (B) and wildtype fish (B′). Microphthalmia and anophthalmia present variably in *gdf6a^s327/s327^* fish (e.g. right and left eyes in B, respectively) and eyes are often noted to possess a lens (L), though in this instance the right eye is inverted such that the anterior segment is oriented towards the midline. RPE (r) and a thin layer of photoreceptors (p) are discernable in *gdf6a^s327/s327^* fish (C′), though other retinal layers are not recognizable due to multiple tissue infoldings. In panel D, the lens was presumably displaced away from the iris during dissection/fixation. Note C is at higher magnification compared to D. Scale bar in B 1 mm; C, D is .5 mm; C′, D′ is .1 mm. L, lens; v, vitreous; r, RPE layer; p, photoreceptor layer.

**Figure 2:**
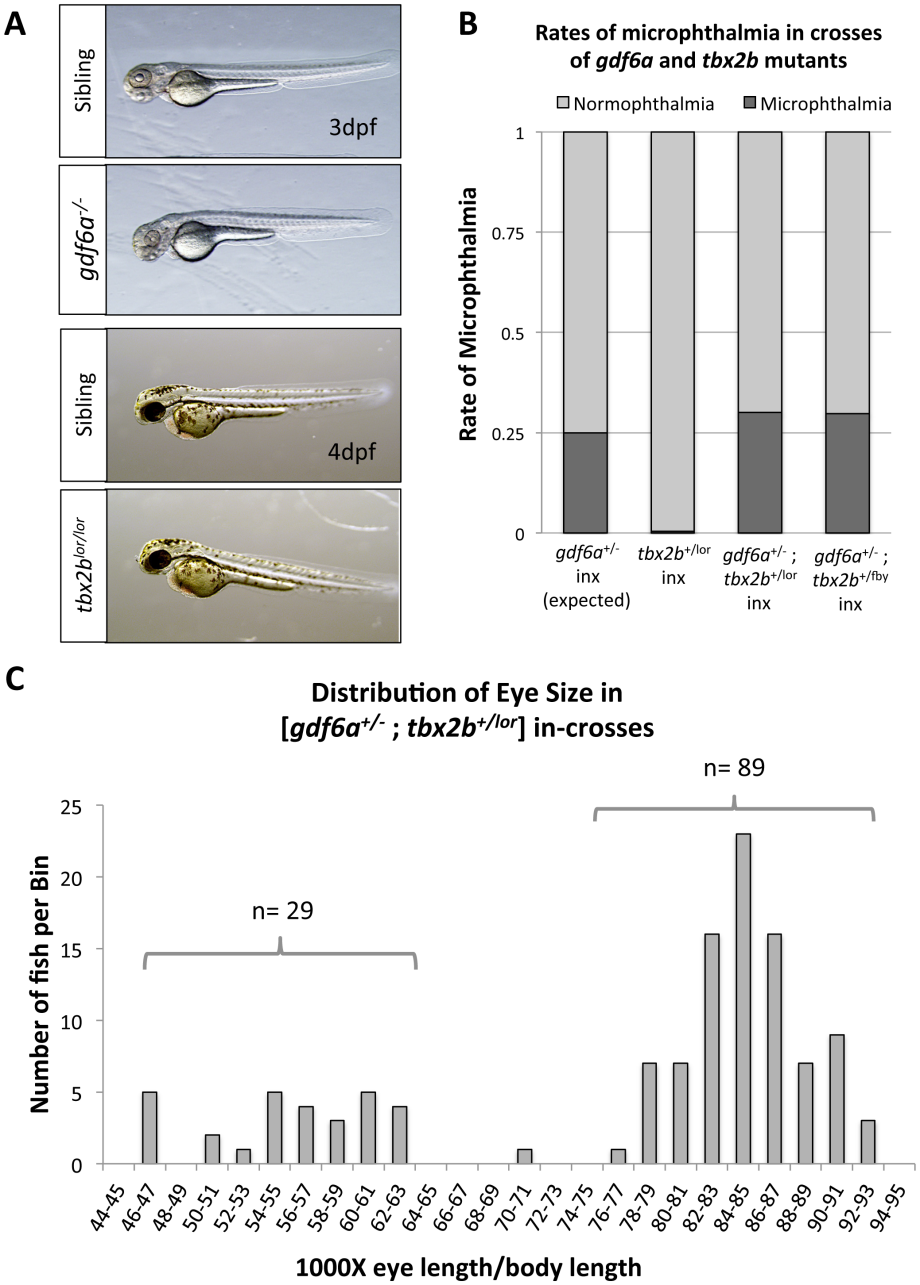
Disruption of *tbx2b* does not modify the *gdf6a* microphthalmic phenotype. **A**. *gdf6a^s327/s327^* mutants exhibit microphthalmia (observed at 3dpf) but *tbx2b^−/−^* mutants (*lor* and *fby*) do not, indicating that disruption of *tbx2b* does not interfere with identical pathways as *gdf6a* in early eye development. **B**. Microphthalmia is rarely observed in *tbx2b* mutant in-crosses (inx) alone (*tbx2b^+/lor^* in-cross shown, n = 220) compared to in-crosses of *gdf6a^+/s327^*, which yield 25% with microphthalmia (following Mendelian ratios of inheritance and recessive phenotype). When [*gdf6a^+/s327^;tbx2b^+/lor^*] or [*gdf6a^+/s327^;tbx2b^+/fby^*] compound heterozygous mutants are in-crossed (n = 121 and 195 respectively, both at 6dpf), rates of microphthalmia do not increase significantly from rates expected of in-crosses of *gdf6a^+/s327^* alone (*X* ^2^ p = 0.873 and p = 0.137, respectively). **C**. The eye size compared to body length (shown as ratio) of a [*gdf6a^+/s327^;tbx2b^+/lor^*] in-cross does not reveal a subset of intermediate eye sizes, but remains bimodal, with the normophthalmic curve (right curve) showing a normal distribution (Shapiro-Wilk Normality test, W = 0.9888, p = 0.6532) (n = 118, 4dpf).

Microphthalmia is apparent in homozygous *gdf6a^s327/s327^* larvae by 3 days post-fertilization (dpf), but is not observed in mutants for *tbx2b^lor/lor^* or *tbx2b^fby/fby^* through any age (Figure 1A, Figure 2A). Microphthalmic eyes in *gdf6a^s327/s327^* fish persist to adulthood, exhibiting variably small eyes (or none at all), while eyes in *tbx2b* mutants develop normally. Paraffin sections demonstrate the microphthalmic eyes as they are positioned in the heads of *gdf6a^s327/s327^* adult fish, with developed but irregularly-shaped lens and a lack of retinal lamination (Figure 1B, C, D), consistent with our recent studies [43].

Concerted disruption of both genes is the most sensitive test of the hypothesis that *gdf6a* and *tbx2b* share a genetic interaction in the early stages of eye development. *Gdf6a^s327/s327^* and *tbx2b^lor/lor^* (or, where noted in figures, *tbx2b^fby/fby^*) mutants were crossed to produce compound heterozygous, [*gdf6a^+/s327^*;*tbx2b^+/lor^*] mutants, which were then in-crossed to procure a full range of genotypic combinations, including compound homozygous mutants. The proportion of resulting offspring exhibiting microphthalmia did not significantly deviate from 25%, suggesting that mutation in *tbx2b* does not affect the rate of microphthalmia in a *gdf6a^+/s327^* background (Figure 2B). Genotyping these fish revealed that every microphthalmic larva had a *gdf6a^s327/s327^* genotype, and all combinations of *tbx2b* alleles (*tbx2b^lor^* and wildtype alleles) existed among these microphthalmic larvae, supporting the null hypothesis that *gdf6a* mutation independently causes microphthalmia in the compound mutants. The eye size-to-body ratio of this pool was assessed to detect possible changes in phenotype severity. Both a microphthalmic population and a normophthalmic population were present and distinct from each other (Figure 2C). The values in the normophthalmic population were statistically normal in their distribution (Shapiro-Wilk Normality test, W = 0.9888, p = 0.6532), further arguing against multiple populations of eye size being present. For confirmation, the eye-to-body ratios were also measured in an in-cross of [*gdf6a^+/s327^*;*tbx2b^+/fby^*] mutants, with similar results (Figure S3B). Only a single population of eye size was observed in in-crosses of *tbx2b^+/lor^* mutants (Figure S1A). We did not explicitly test if combined mutations affected the *severity* of the microphthalmic phenotype (though no such difference is obvious in the data), because the manner in which data was collected did not support that analysis (we lacked foresight whilst collecting data to anticipate comparing between clutches of fish, which would require identical husbandry and timing of dechorionation: eye and body length are not allometric), though the data regarding *rate* of phenotype (from within a clutch) is robust and similar between all genotypes. We conclude that although *gdf6a* regulates *tbx2b* expression during early eye morphogenesis [45], they do not genetically interact to cause microphthalmia in any obvious manner.

### *gdf6a* regulation of cone differentiation differs from predictions derived from phenotypes of *tbx2b* mutants

*Tbx2b* is proposed to regulate UV cone-versus-rod fate, based on homozygous *tbx2b^lor/lor^* and *tbx2b^fby/fby^* mutants which present with a paucity of UV cones and an excess of rod photoreceptors [11]. Because *tbx2b* is downstream of *gdf6a* during the previously examined stages of retinal development, we hypothesized that the two genes may share a genetic pathway in a likewise fashion during photoreceptor development. This hypothesis predicts that UV cone and rod development should be disrupted in *gdf6a^s327/s327^* mutants, similar to observations in *tbx2b^−/−^* mutants.

**Figure 3:**
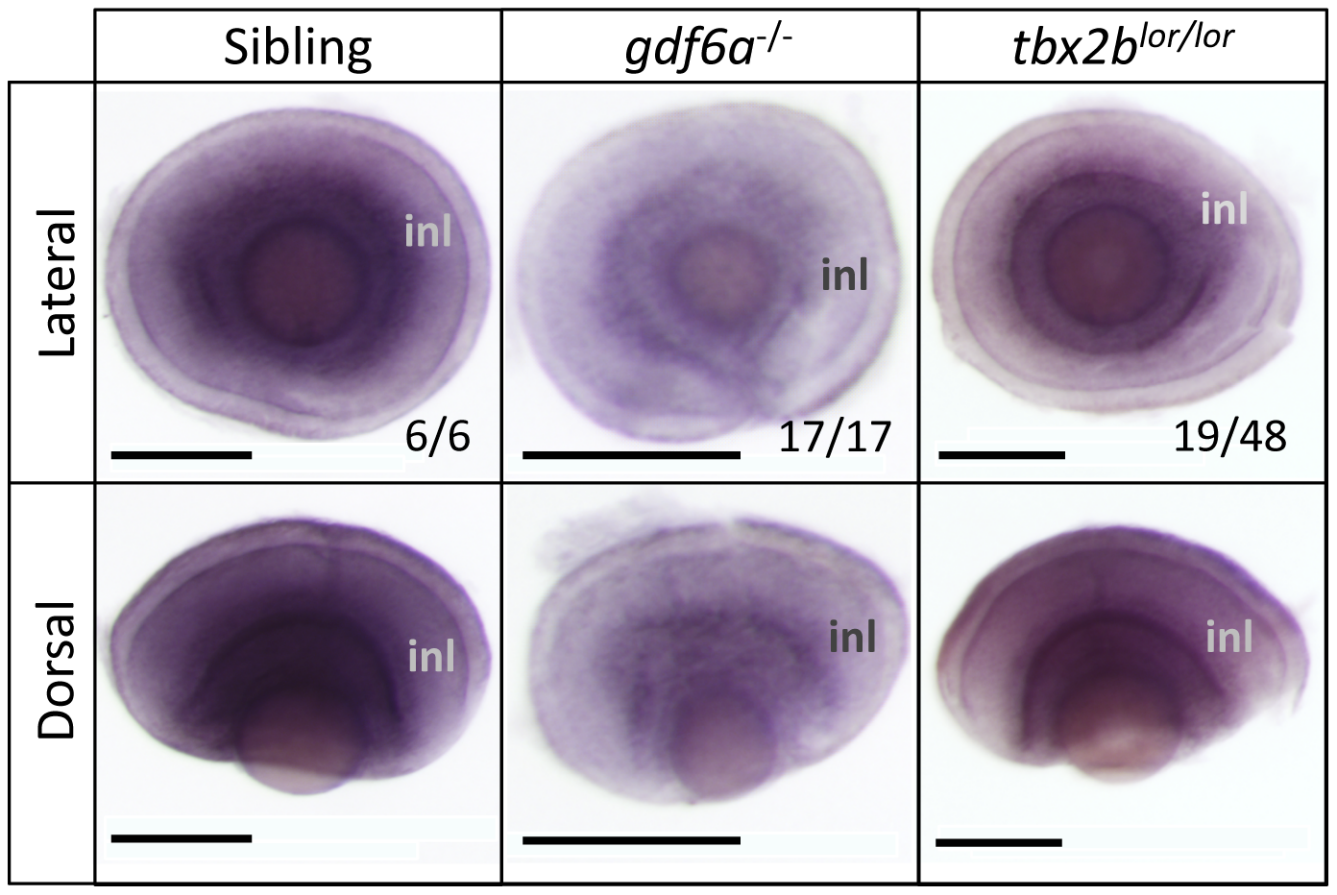
*gdf6a* positively modulates the abundance of *tbx2b* transcript during stages of retinal development when photoreceptors differentiate. All panels show in situ hybridization using *tbx2b* riboprobe. *gdf6a^s327/s327^* mutants have less tbx2b expression at 3 days post-fertilization (dpf) compared to normophthalmic siblings, akin to tbx2blor/lor mutants. Fractions represent proportion of clutch represented by image shown. Scale bars 100 μm; inl, inner nuclear layer.

Abundance of *gdf6a* expression during early ocular morphogenesis correspondingly affect expression levels of *tbx2b* [45]. We investigated whether this direct relationship still held later on in development, when the eye is developed and photoreceptors are assuming their respective fates. At 72hpf *gdf6a^s327/s327^* mutants appear to have less *tbx2b* expression in their retinas compared to wild type siblings, similar to the apparently low *tbx2b* expression in *tbx2b^lor/lor^* mutants of the same age (Figure 3). In wild type retina *tbx2b* expression was not excluded from any of the retinal layers, but was perceived to be most abundant in the ganglion cell layer and vitreal half of the inner nuclear layer. Qualitatively, the latter tissue layer was the one with the greatest reduction of *tbx2b* abundance in the *gdf6a^s327/s327^* mutants.

**Figure 4:**
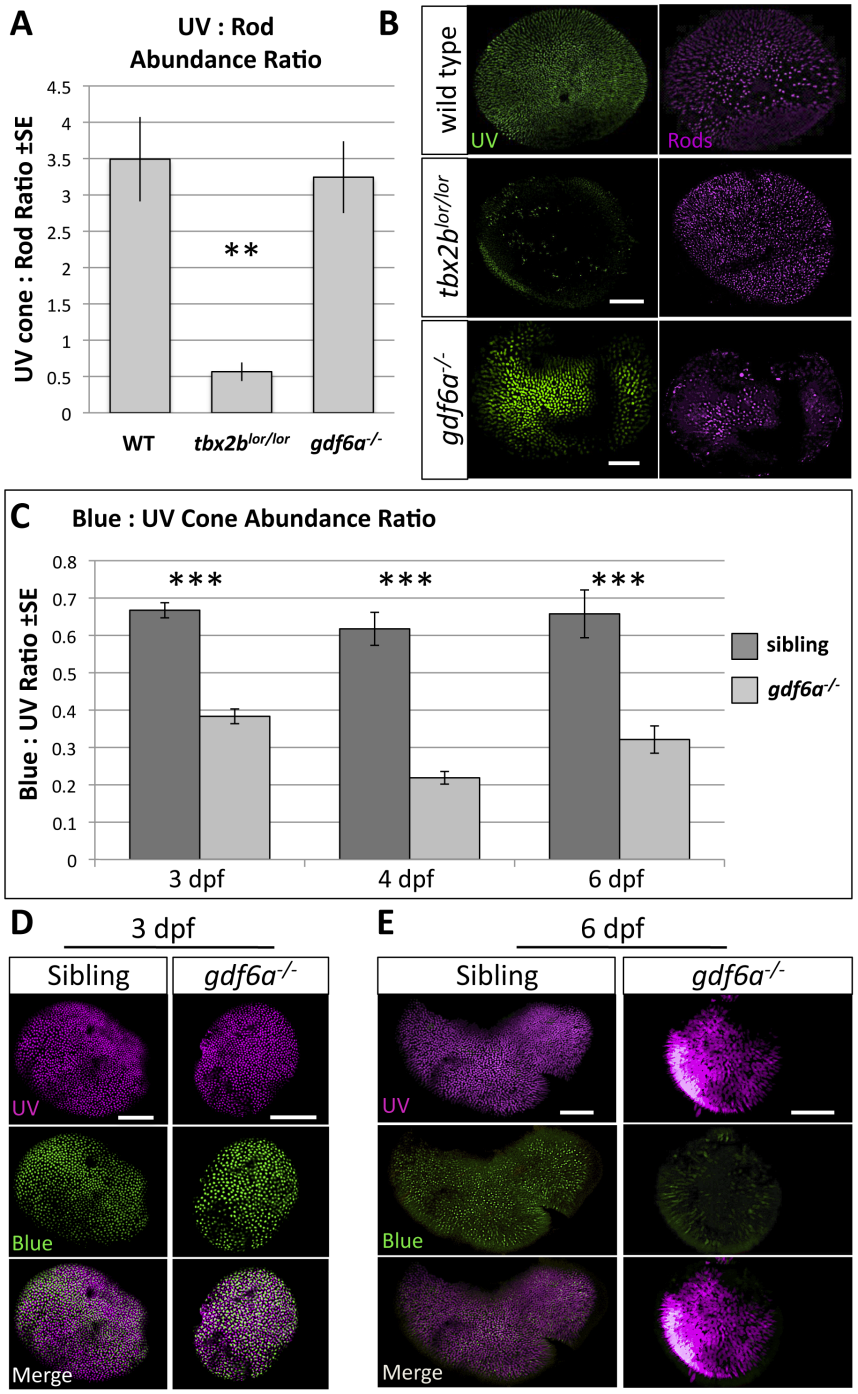
Mutation in *gdf6a* does not disrupt tbx2b function in UV-versus-rod photoreceptor specification, but *gdf6a* rather plays a role in blue cone specification. A,B. *tbx2b^lor/lor^* mutants have fewer UV cones and more rods than wildtype fish (the *lots-of-rods* phenotype) (Kruskall-Wallis ANOVA, **p<0.005), but *gdf6a^s327/s327^* mutants have a normal abundance ratio and distribution of UV cones and rods (n = 10 wildtype, 8 *tbx2b^lor/lor^*, and 7 *gdf6a^s327/s327^*; UV cones expressing GFP and rods were labeled with antibody 4C12). Scale bars 30 μm and 80 μm, respectively. C. Larval *gdf6a^s327/s327^* mutants have a unique cone photoreceptor phenotype in which there are significantly fewer blue cones relative to UV cones at all ages examined (which is not observed in *tbx2b^lor/lor^* or *tbx2b^fby/fby^* mutants- not shown) (Kruskall-Wallis ANOVA, ***p<0.001) Sample sizes at 3 days post-fertilization (dpf) are n = 17 larvae per genotype quantifying cells visualized via opsin in situ hybridization (Panel D); at 4 dpf data are from n = 9 wild type and n = 13 mutants assessed via GFP and mCherry transgene expression in cones; at 6dpf data are from 2 replicates of n = 4+7 wild type and n = 5+6 mutants assessed via transgene expression in cones (Panel E). D. UV and blue cones identified in 3 dpf by in situ hybridization against their respective opsins (Scale bars are 100 μm). E. UV and blue cones identified in transgenic lines at 6dpf by expression of GFP and mCherry, respectively (Scale bars are 60 μm and 40 μm in sibling and mutants, respectively).

We examined UV cone and rod photoreceptors in *gdf6a^s327/s327^* larvae and, contrary to our expectations, they did not show a *lots-of-rods* phenotype; UV cones and rods in microphthalmic *gdf6a^s327/s327^* eyes had normal relative abundance and distribution compared to wild type and to normophthalmic sibling eyes (Figure 4A, B). Therefore, despite that *gdf6a* appears to regulate the abundance of *tbx2b* transcript during times of development when photoreceptors are specified (Figure 3), disrupting *gdf6a* alone does not recapitulate phenotypes observed from disrupting *tbx2b* with regards to UV cone-versus-rod fate. This suggests that the downstream reductions in *tbx2b* resulting from mutation of *gdf6a* are insufficient in magnitude, or too different in their timing, to measurably produce effects upon UV cone or rod photoreceptor cell fate.

Assessing these phenotypes using a second metric was warranted because microphthalmia, a defect in early organogenesis, could have confounded potential differences in rod:cone ratios that are established later in development. Thus the abundance of UV cones in 6dpf *gdf6a^s327/s327^* larvae was also compared to the abundance of blue cones. We counted the number of UV and blue cones expressing GFP and mCherry, respectively, in transgenic fish (see Methods). This provided confirmation that UV cones were not reduced in abundance. However, the number of cones of the blue spectral subtype was dramatically reduced and often distributed in a patchy pattern in *gdf6a^s327/s327^* retinas compared to normophthalmic siblings (Figure 4C, E). It was of interest to determine if this difference between genotypes was observable earlier in development, when cones are first detectable. Quantifying relative cone abundance in these transgenic fish at 4 dpf confirmed the difference between genotypes arises early (Figure 4C). This was further confirmed at 3 dpf after identifying cones using UV and blue opsin riboprobes via *in situ* hybridization (Figure 4C, D). This suggests that *gdf6a* signaling alone does not regulate cone-versus-rod development in the same fashion that *tbx2b* does, but instead appears to be acting in the differentiation of cone spectral subtypes.

### Specificity and utility of rat monoclonal antibody 10C9.1 for labeling UV cones

To better detect UV cones and phenotypes of interest, we isolated a novel monoclonal antibody raised in rat and characterized its ability to specifically detect UV-sensitive opsin (product of *opn1sw1*, ZFIN ZDB-GENE-991109-25). The clone giving rise to antibody 10C9.1 was derived from a rat injected with a peptide antigen equivalent to the 20 amino-terminal amino acids of trout UV opsin (Figure S2E), and polyclonal sera from this rat had previously been shown to label UV cones in trout [34] and zebrafish [16]. 10C9.1 was isotyped to an IgG2c.

**Figure 5:**
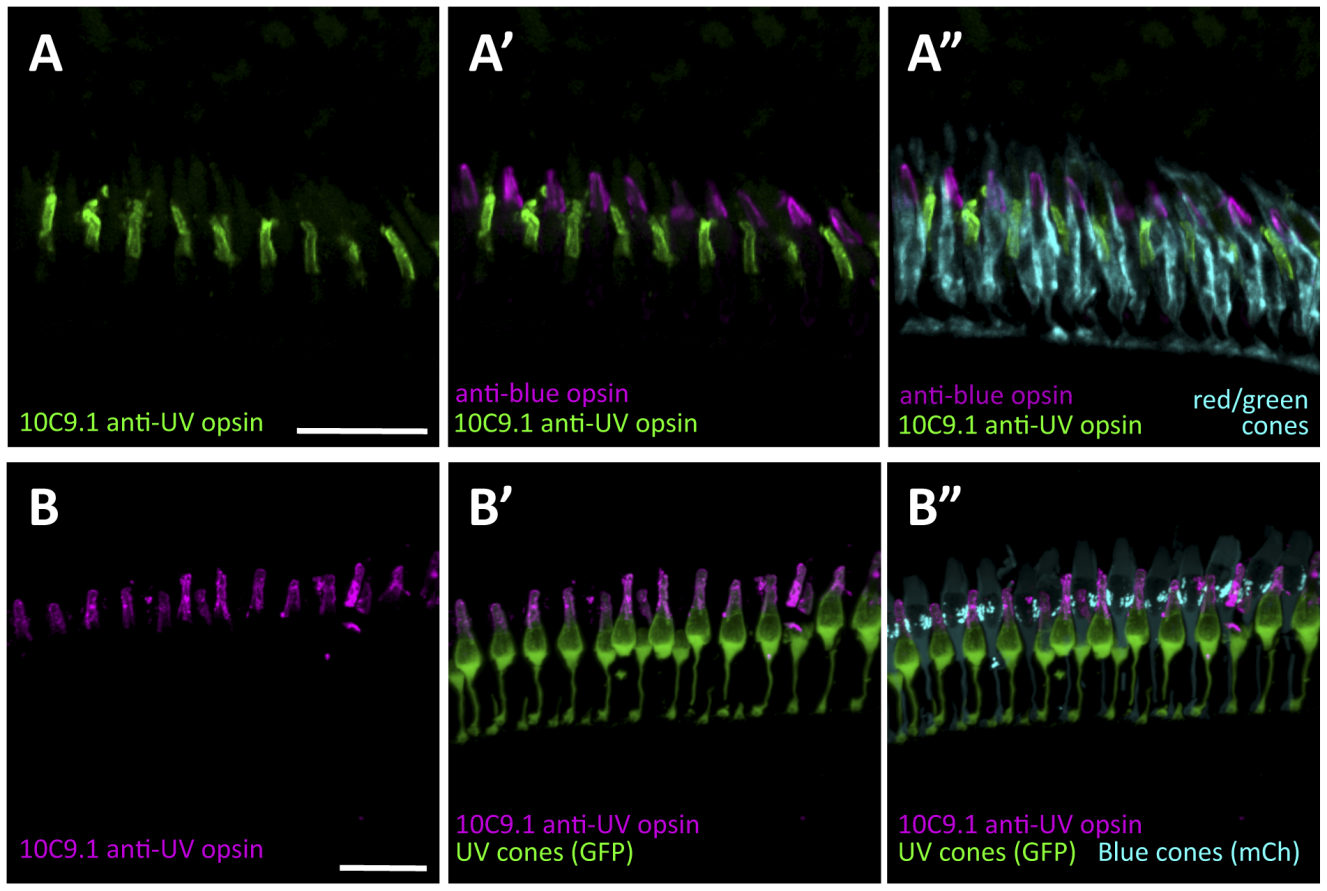
A monoclonal antibody raised in rat (10C9.1) labels zebrafish UV cone outer segments, allowing all cone subtypes to be simultaneously labeled by immunohistochemistry. Antibody 10C9.1 specifically labels the outer segments of a class of short single cones in the adult zebrafish retina (Figure S1 panel A). 10C9.1 specificity is supported (see Figure S1 panels B–D), including by a dramatic decrease in number of cells labeled when 10C9.1 is applied to retinas from zebrafish mutants (*tbx2b^lor/lor^*) that have a paucity of UV cones. A. The population of single cones labeled by 10C9.1 is the UV cones, because established antibodies against the other single cone class, the blue cones, labels a distinct cone population (A′). 10C9.1 enables an unprecedented combination of antibodies raised in different species that simultaneously label and distinguish all cone photoreceptor subtypes (A”). E. Further evidence that 10C9.1 labels UV cone outer segments comes from its co-localization with UV cones filled with green fluorescent protein (GFP), and its exclusion from blue cones filled with mCherry (mCh) in transgenic zebrafish (*Tg(-5.5opn1sw1:EGFP)kj9;Tg(-3.5opn1sw2:mCherry)ua3011*). Panel B is available as Movie S1. Scale bars 30 μm.

Application of 10C9.1 to cryosections of adult zebrafish retina, and counterstaining of lipid-rich material using BODIPY-TMR, revealed that 10C9.1 labels the outer segments of photoreceptors with a single cone morphology (Figure S2A). Restriction of the labeling to the cone outer segment is consistent with labeling of an opsin because in healthy eyes opsins are abundant only in this cellular compartment. Considering UV cones are one of only two spectral subtypes that exhibit a single cone morphology (along with blue cones, and in contrast to the green and red cones that are fused into a double cone morphology), the labeling also suggested that 10C9.1 was detecting either UV or blue cones. Simultaneous labeling using 10C9.1 and a well-characterized anti-blue opsin antibody [21] demonstrated that 10C9.1 labels a population of single cone photoreceptors that is distinct from the blue cones (Figure 5A). Further, the 10C9.1 labeling was localized to cone cells with a short single cone morphology with the outer segments in a more vitreal (basal) position than that of blue cone outer segments (Figure 5A), and this is exactly consistent with the morphology of UV cones as identified via *in situ* hybridization [19], [51], [52], immunohistochemistry [21], and microspectrophotometry [16], [17]. Simultaneous labeling of 10C9.1 and zpr1 antibody that labels the entire plasma membrane of red/green double cones demonstrated no apparent overlap in labeling (Figure 5A).

**Figure 6:**
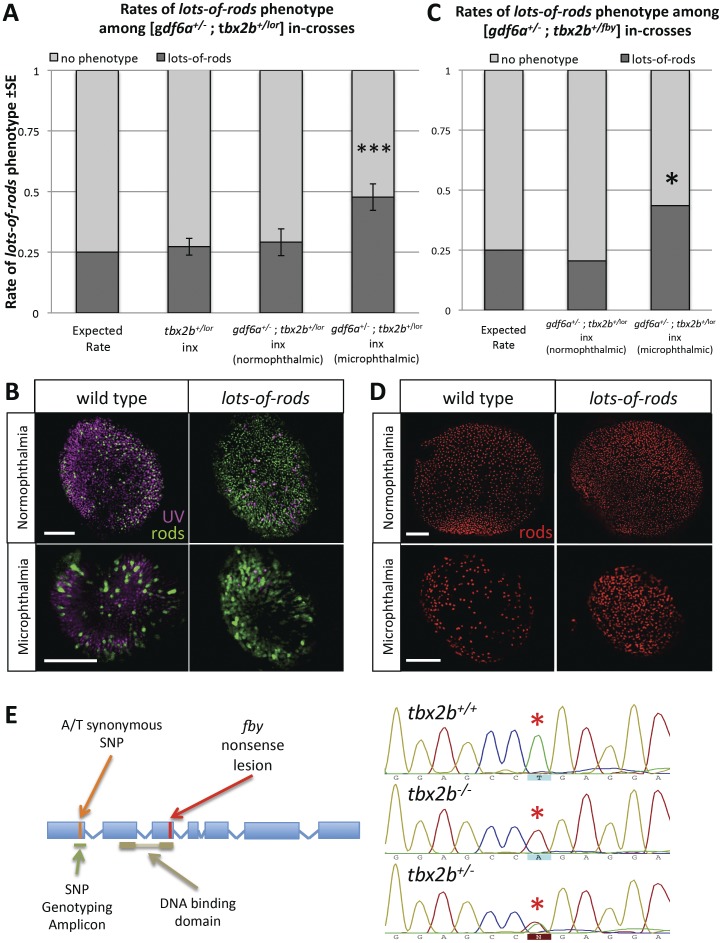
*gdf6a* modulates *tbx2b* regulation of UV cone and rod development. **A, B**. When [*gdf6a^+/s327^;tbx2b^+/lor^*] compound heterozygous mutants are in-crossed (inx), a disproportionate fraction of microphthalmic offspring exhibit the *lots-of-rods* phenotype compared to normophthalmic siblings, *tbx2b^+/lo^* ^r^ in-crosses, and to predicted Mendelian ratios of the recessive *lots-of-rods* phenotype (*X* ^2^ ***p<0.001; 3 replicates of n = 17, 19, 35 microphthalmics; 6dpf). UV cones and rods were labeled using antibodies 10C9.1 and 4C12 displayed in magenta and green, respectively. A portion of microphthalmic larvae with the *lots-of-rods* phenotype has a *tbx2b^+/lor^* genotype (see Table 1). **C, D**. When [*gdf6a^+/s327^*;*tbx2b^+/fby^*] compound heterozygous mutants are in- crossed, the *lots-of-rods* phenotype is again observed at higher rates in microphthlamic eyes compared to normophthalmic eyes (*X* ^2^ *p = 0.007; 1 replicate, n = 39 microphthalmics, 6 dpf). Panel D shows rod opsin in situ hybridization (red). Scale bars are all 50 μm. **E**. Genotyping for the *lor* mutation was performed via linkage analysis using an A/T synonymous SNP located before the DNA binding domain of *tbx2b* in *lor* and non-*lor* alleles, respectively. *gdf6a^s327/s327^* mutants with a corresponding SNP of T were used in crossing of the mutant lines.

The specificity of antibody 10C9.1 was assessed using two additional strategies. First, we noted that no such labeling was apparent when the 10C9.1 primary antibody was excluded or when it was substituted by another rat IgG2c primary antibody (Figure S2C). Second, we noted that very few cells were labeled when 10C9.1 was applied to retinas that are known to have few UV cones, i.e. retinas from adult *tbx2b^lor/lor^* mutants (Figure S2C, see also top row of Figure 6B for the same approach on retinas from normophthalmic larvae).

We further confirmed that 10C9.1 is labeling UV cones by its co-localization with an established anti-UV opsin antibody raised in rabbit [21] (Figure S3). Finally, we applied 10C9.1 to adult double transgenic zebrafish that we recently characterized as expressing GFP throughout their UV cones and expressing mCherry throughout their blue cones *Tg(-5.5opn1sw1:EGFP)kj9;Tg(-3.5opn1sw2:mCherry)ua3011* [33], [53], [54]. This labeling again revealed localization of 10C9.1 exclusively to the outer segments of UV cones (Figure 5B and Movie S1).

In sum, the specificity of the antibody 10C9.1 for labeling UV cones was determined by its localization to the expected cellular compartment (photoreceptor outer segment) and only within the cone cells of the expected morphology (short single cones); this was complemented by co-localization of 10C9.1 with well-characterized antibodies and transgenes that label UV cones, and exclusion from similar markers that label other cones. Further, 10C9.1 labeling was greatly reduced when applied to retinas with few UV cones. The utility of 10C9.1 is enhanced by being a stable monoclonal source of reagent. Further, because the host animal was rat, 10C9.1 can be used in multi-label experiments with the large selection of available antibodies raised in rabbit and mouse. The latter includes that one can, for the first time we are aware of, simultaneously distinguish each of the cone subtypes of zebrafish using immunohistochemistry (Figure 5A”). 10C9.1 is available from the corresponding author or from Immunoprecise Antibodies Inc (Victoria BC, Canada www.immunoprecise.com; antibody name “UVop-10C9.1”).

### A subtle interaction between *gdf6a* and *tbx2b* modulates the *lots-of-rods* phenotype

Based on the lack of a *lots-of-rods* phenotype in *gdf6a^s327/s327^* mutants (Figure 4A, B), it had appeared that *gdf6a* might not regulate cone-versus-rod development. However, a more sensitive test to detect the presence of an interaction is concerted disruption of both genes. To determine whether *gdf6a* and *tbx2b* interact in photoreceptor development, we examined UV cone and rod photoreceptors in the progeny of compound heterozygous [*gdf6a^+/s327^*;*tbx2b^+/lor^*] in-crosses (and [*gdf6a^+/s327^*;*tbx2b^+/fby^*] in-crosses). We hypothesized that if there were a less linear, subtler interaction between *gdf6a* and *tbx2b*, disrupting both genes simultaneously would reveal it, by resulting in a synergistic phenotype or occurrence of phenotypes among larvae whose genotypes predict none.

Microphthalmic eyes from the aforementioned in-crosses showed an elevated rate of the *lots-of-rods* phenotype (48% of microphthalmic eyes showed a *lots-of-rods* phenotype among [*gdf6a^+/s327^*;*tbx2b^+/lor^*] in-crosses, a significantly greater proportion than the predicted 25%; *Χ* ^2^ p<0.001). This contrasted eyes from normophthalmic siblings wherein the rate of the *lots-of-rods* phenotype (29%) did not statistically differ from the expected Mendelian rate (Figure 6A, B). The elevation in *lots-of-rods* phenotypes among microphthalmic larvae also differed significantly from rates in *tbx2b^+/lor^* in-crosses (without *gdf6a* mutation), where 27% of embryos exhibited the *lots-of-rods* phenotype, which was also statistically consistent with predicted Mendelian inheritance (Figure 6A).

To better define the genetics of this system, we repeated the in-crosses of *gdf6a^−/−^* and *tbx2b^lor/lor^* mutants in a fashion that allowed us to track genotypes via single nucleotide polymorphisms (SNPs) within the *tbx2b* gene. The use of SNPs was necessitated by the fact that the *tbx2b^lor^* mutation remains undefined. Screening a panel of potential SNPs (see Methods) on several fish of each genotype allowed us to identify homozygous *tbx2b^lor/lor^* and *gdf6a^s327/s327^* founders wherein each had a different homozygous SNP in the *tbx2b* gene (in the first exon, Figure 6E). These were bred to generate compound heterozygous fish [*gdf6a^+/s327^*;*tbx2b^+/lor^*] within which we could reliably track the inheritance of the *tbx2b^lor^* allele.

To test if the elevated rates of the *lots-of-rods* phenotype accords with a partial loss of *tbx2b* function, as predicted from a genetic interaction, 35 microphthalmic larvae from the aforementioned in-cross were genotyped. All larvae were *gdf6a^s327/s327^*, as expected from their microphthalmic phenotype. Amongst these, we expected a 25% (i.e. 9/35 larvae) rate of the *lots-of-rods* phenotype based on a recessive pattern of inheritance (see “Expected” column in Table 1). We also expected all 9 of these larvae to have a *tbx2b^lor/lor^* genotype through SNP analysis. But we observed the previously mentioned increase in rate of the *lots-of-rods* phenotype −13/35 or 37% of the larvae had the phenotype (“Observed” column in Table 1). Genotyping revealed that 4 of these 13 *lots-of-rods* phenotypic larvae were, in fact, heterozygous *tbx2b^+/lor^* (unexpected), whereas 9 were homozygous *tbx2b^lor/lor^* (matching the expected rate of homozygosity). This indicates that the *lots-of-rods* phenotype can occur in a subset of heterozygous *tbx2b^+/lor^* fish, but only when both copies of *gdf6a* are mutated. Genotyping also revealed that normophthalmic individuals with the *lots-of-rods* phenotype were all homozygous *tbx2b^lor/lor^*. Therefore, the slightly elevated rate of the *lots-of-rods* phenotype (29%, not significantly different from Mendelian 25%, see above) in these normophthalmic eyes likely resulted from reduced survival of other genotypes. This might be expected if toxic mutations were not yet bred out following random chemical mutagenesis.

**Table 1:** Identification of compound [*gdf6a^s327/s327^*; *tbx2b*] mutants with mismatched phenotype and genotype regarding *tbx2b*.

		EXPECTED				OBSERVED			
Phenotype:		wild type	lots-of-rods			wild type	lots-of-rods		
	Raw# /35 total	26	9			22	13		
	%	75%	25%			63%	37%		
			lots-of-rods				lots-of-rods		
*tbx2b* Genotype:			+/+	+/−	−/−		+/+	+/−	−/−
	Raw# /35 total		0	0	9		0	4	9
	%		0%	0%	**25%**		0%	**12%**	**25%**

Because the rate of *lots-of-rods* among microphthalmic larvae (48%) does not reflect any classical Mendelian ratio that may explain such elevated rates, we examined the effect of the stronger null *tbx2b^fby^* allele in the same context with *gdf6a* mutation, with the suspicion that introducing a null *tbx2b* mutation may induce a more severe phenotype. An in-cross of [*gdf6a^+/s327^*;*tbx2b^+/fby^*] showed similar results to [*gdf6a^+/s327^*;*tbx2b^+/lor^*] in-crosses, with the *lots-of-rods* phenotype occurring in 44% of microphthalmic eyes again significantly (*Χ* ^2^ p = 0.007) more than normophthalmic siblings (20.5%) (Figure 5C, D).

## Discussion

Efforts to model vision regeneration using stem cells are stymied by the difficulties procuring progenitors for cone photoreceptors, the cells required for daytime vision, in established murine models. One of the obstacles in this respect is a limited knowledge of the genetic regulation of cone development from retinal progenitor cells. To complement and utilize our novel zebrafish cone regeneration model [33], we are investigating candidate regulatory factors of cone and cone subtype development. In this paper, we explored the potential interaction between two genes with recently realized connections to photoreceptor development and degeneration. To this end we demonstrated that these genes, *gdf6a* and *tbx2b*, unexpectedly regulate development of spectral subtypes of cones and interact in the development of UV cones and rod photoreceptors specifically.

*Tbx2b* is one of the most recently recognized regulatory genes directing cone and rod differentiation [11]. *GDF6* (and its homolog *gdf6a*) was selected as a candidate regulator of cone development because deficits in its function cause photoreceptor degeneration, as identified through panels of LCA patients, complemented by murine and zebrafish models [43]. These developments, in synergy with the established regulatory relationships between *gdf6a* and *tbx2b* in early retinal development [45], [50], led us to speculate that *gdf6a* signaling also regulates cone photoreceptor development.

### *Gdf6a* signaling has a conserved role in ocular morphogenesis that does not appear to depend on *tbx2b* activity

The genetic interactions between *gdf6a* and *tbx2b* in zebrafish eye development are not as linear as we had assumed. Although *Tbx2* knockout mice display microphthalmia [41], akin to *gdf6a* loss-of-function models in various vertebrate homologs [43], [50], [51], [52], [53], and *gdf6a* signaling has been previously demonstrated to positively regulate *tbx2b* expression during ocular morphogenesis [45], our data indicate that in zebrafish disruption of *tbx2b* is not sufficient to augment the pathology of microphthalmia observed upon *gdf6a* disruption. This was revealed both by the lack of a microphthalmic phenotype upon *tbx2b* loss of function, and the lack of change in rate or apparent severity of microphthalmia when *gdf6a* and *tbx2b* null mutations were combined. This data ruled out an alternative explanation for the genetic interdependence we observed regarding photoreceptor development, demonstrating that alterations in microphthalmia cannot explain the increased rates of photoreceptor-related phenotypes we noted during concerted gene disruption. Towards a broader relevance of this data, *gdf6a* and *tbx2b* have both been demonstrated to play roles in cell proliferation and establishing dorsal retina identity, though in different animal models [40]–[42], [45], [47], [50], [59]–[62]. Our observations of *tbx2b* disruption in zebrafish contrast that of the mouse homolog *Tbx2*, which is also downstream of ocular BMP signaling, and mutations in this pathway produce a microphthalmic phenotype in mice [41]. It may be that *tbx2b* and *Tbx2* do not share the same role in early retinal development, that *tbx2b* has different spatiotemporal kinetics, or that redundancy with other genes (*tbx2a*, *gdf6b* or others) can compensate in zebrafish.

### Differential role for *gdf6a* amongst the spectral subtypes of cone photoreceptors

We had speculated that *gdf6a* mutants would present with phenotypes similar to *tbx2b* mutants (lots of rods and few UV cones). This speculation was borne upon observations that *tbx2b* specifies rod versus UV cone fate, and *gdf6a* is a positive upstream regulator of *tbx2b* expression. Instead, the data revealed that gdf6a mutants do not exhibit the anticipated phenotype; rather, our observations indicate that *gdf6a* signaling promotes development or maintenance of blue-sensitive cones. This is uniquely promising, suggesting a set of novel regulatory actions in a stage of photoreceptor development that has not been adequately explored in zebrafish before: that of cone opsin spectral *subtype* specification. It is not yet clear whether *gdf6a* serves a role in cone specification, differentiation and/or survival, but one avenue of investigation will be modulating *gdf6a* signaling during proliferation and cone photoreceptor differentiation as replicated in our regeneration model [33]. Pursuing *gdf6a* effects in the regenerative context is especially intriguing since recent work shows that the proliferative response in Müller glia requires regulation of TGFβ signaling [63].

It remains undetermined the extent to which the blue cone-specific requirement for *gdf6a*, established herein, is mechanistically similar to the apparent UV cone-specific requirement of *tbx2b* [11]. At the cellular level, a notable difference in phenotypes is that *tbx2b* mutants present with an excess of rod photoreceptors [11], which we did not observe in *gdf6a* mutants. With respect to cellular sites of action for these genes in determining cell fate, it is not clear whether UV and blue cones are products of a common pool of progenitor cells; although UV cones and blue cones are together the last photoreceptor types to differentiate during retinal development (based on the sequential appearance of detectable opsin transcript in goldfish [64]), the terminal divisions seem to rely upon separate/dedicated progenitor pools [39]. Regardless, it is tempting to speculate that common molecular signaling pathways might lead to a low abundance of blue and UV cones, in *gdf6a* and *tbx2b* mutants respectively, especially considering the epistatic relationship of these genes during both early and late retinal development (see below).

### *Gdf6a* and *tbx2b* genetic interdependence in UV cone and rod photoreceptor differentiation

Considering the disparate ocular phenotypes observed between their respective mutants, during both early and late retinal development, the genetic interaction between *gdf6a* and *tbx2b* is neither simple nor linear. But interpreting this relationship is made more complex by the low UV cone abundance and high rod abundance (the *lots-of-rods* phenotype) observed in high rates among compound mutant larvae. Some of these larvae were found to display the recessive *tbx2b* phenotype despite being genetically heterozygous for the *tbx2b* mutation (which alone does not yield the *lots-of-rods* phenotype). *Gdf6a* appears to modulate *tbx2b* indirectly, suggestive of a genetic interdependence in a UV cone fate decision. One hypothesis to this end is that *gdf6a* and *tbx2b* both promote a common activity, perhaps via being in the same pathway (as per Figure 3). Thus a lack of *gdf6a* signaling would reduce the efficacy of *tbx2b* to promote a UV cone fate, causing the assumption of a *lots-of-rods* phenotype or a wild type phenotype to become more random. Alternatively, *tbx2b* expression may have a minimum threshold for activity that is sensitive to perturbations (including disrupted *gdf6a* signaling) affecting *tbx2b* mRNA transcript levels. Alternatively, *gdf6a* signaling may alter the timing of cell cycle exit of photoreceptor progenitors, as has been established for *gdf11* [65]; thus with altered *gdf6a* there may be increased probability that progenitors undergo specification/differentiation and invoke *tbx2b* expression at an inappropriate time, thereby shifting cell fates.

## Conclusion

Further exploration into pathways utilizing *tbx2b* and *gdf6a* would clarify the order or pattern of photoreceptor specification, which will also provide insight into zebrafish cone mosaic formation and photoreceptor regeneration following injury. Zebrafish possess latent stem cells within the retina and have a robust neural regenerative capacity. Because of these properties, zebrafish are a promising *in vivo* model to study not just photoreceptor development, but also regulation of cone photoreceptor regeneration and integration. With zebrafish as an impressive model of functional regeneration, stem cell therapy for restoring daytime vision can become a reality.

## Methods

### Ethics statement

Fish care and protocols were approved by the Animal Care and Use Committee: Biosciences at the University of Alberta. Rat care and protocols were approved by the Animal Care Committee at the University of Victoria. In each instance protocols and care were in accordance with the Canadian Council on Animal Care.

### Animal care and establishment of mutant crosses

Zebrafish (*Danio rerio*) were raised and maintained according to standard procedures [66]. Larvae were kept at 28°C in E3 media. *Gdf6a^s327/+^* [45] (ZFIN ID ZDB-ALT-050617-10), *tbx2b^p25bbtl/p25bbtl^* (ZFIN ID ZDB-GENO-080920-2, referred in text and figures as *tbx2b^lor/lor^*)[11], and *tbx2b^c144/+^* (ZFIN ID ZDB-GENO-130130-6, referred in text and figures as *tbx2b^fby/+^*) [42], [67] fish were gifted from Andrew Waskiewicz (University of Alberta), James Fadool (Florida State University), and Josh Gamse (Vanderbilt University) respectively. These lines were crossed to create [*gdf6a^+/s327^; tbx2b^+/lor^*] and [*gdf6a^+/s327^; tbx2b^+/fby^*] compound heterozygous mutants, which were subsequently in-crossed to acquire [*gdf6a^s327/s327^; tbx2b^lor/lor^* ] and [*gdf6a^s327/s327^; tbx2b^fby/fby^*] compound homozygous mutants along with siblings of various genotypic combinations. The *gdf6a^s327/s327^* and *tbx2b^lor/lor^* lines were also crossed with transgenic lines: Tg(-5.5opn1sw1:EGFP)kj9 [20] and Tg(-3.5opn1sw2:mCherry)ua3011 [33], [53] expressing fluorescent proteins in UV and blue cones, respectively.

### Assessing phenotypes, genotyping and linkage analysis

Larvae from the above-described mutant lines were assessed for phenotype and, where noted, subsequently genotyped. *Gdf6a^s327/s327^* larvae were identified by their microphthalmic phenotype starting at 3dpf. Where relevant, eye size-to-body length ratios were calculated to check for an intermediate eye size phenotype (details below). To identify putative *tbx2b^lor^* or *tbx2b^fby^* homozygous mutants, larval retinas were removed from the heads, flatmounted, imaged on a Zeiss Axio Observer.Z1 microscope with AxioCam software (Carl Zeiss MicroImaging, Oberkochen), and thereby screened for the *lots-of-rods* phenotype, characterized by an abnormally large population of rod photoreceptors and a small population of UV cones, which is exacerbated in *tbx2b^fby/fby^* mutants [11]. Heterozygous mutant and wildtype *gdf6a* and *tbx2b* siblings, which do not have a phenotype, were identified by genotyping.

Genomic DNA was isolated as described by Meeker et al. [68]. Genotyping for *gdf6a^s327^* was done by restriction fragment length polymorphism (RFLP) analysis. Primers designed by Gosse and Baier [45] amplify a 280 bp region including the *gdf6a^s327^* locus. This PCR product was either digested with SfaNI restriction enzyme and run on a gel, or sequenced with a BigDye v3.1 kit (Invitrogen, Carlsbad, Cat. # 4337455) and submitted to Molecular Biology Services Unit at the University of Alberta. *Tbx2b^fby^* was genotyped through RFLP analysis; primers used were designed by Snelson et al. [42] and amplify a 318 bp region featuring the *tbx2b^fby^* locus. The PCR product was digested with MseI restriction enzyme and run on a gel.

The lesion of the *tbx2b^lor^* allele has been linkage-mapped to the region of *tbx2b* [11], but has not yet been identified. Therefore, genotyping fish for *tbx2b^lor^* required developing a single nucleotide polymorphism (SNP) genotyping assay, and inferring the *tbx2b* genotype. We explored six SNPs annotated in the zebrafish *tbx2b* gene in the *Ensembl* database; using Geneious software, we designed primers to amplify each of the SNPs to genotype via sequencing (performed at Molecular Biology Service Unit, University of Alberta) (Table 2). Each SNP was amplified from representative adult male *tbx2b^lor/lor^* fish, and from representative adult female *gdf6a^s327/s327^* fish, and examined for homozygosity at each SNP. While several homozygous SNPs were identified, only two were different between the two populations, and one synonymous SNP (bold text in Table 2) was chosen based on the reliability of PCR amplification and sequencing (Figure 6E). Thus, the presence of an “A” in this SNP implied inheritance of the parental *tbx2b^lor^* allele, while presence of a “T” implied the parental WT allele (in fish with mutation in *gdf6a*). These adult fish were crossed, and all resulting [*gdf6a^+/s327^*; *tbx2b^+/lor^*] fish were confirmed to be heterozygous at the relevant SNP. In-crossing these compound heterozygotes and genotyping the resultant normophthalmic progeny confirmed that the SNP genotyping assay consistently predicts the lots-of-rods phenotypes.

**Table 2:** Primers used to identify single nucleotide polymorphisms (SNPs) for *tbx2b* genotyping.^1^

SNP:	FWD primer	REV primer	Size (bp)
rs40785418	TGC GCT TGA ATG GAC ATC CGC A	AAG GCG AGA GCA GAC AGC GG	196
**rs40952575**	**CGG ACC ATA CCC TGG CCG GA**	**TGG TCC CAT AGA TCC TTC GCT TCC A**	**160**
rs41094432	CTG CCG ACG ACT GCC GCT AC	TCC CCA GTA GCT GGG CTA TCC G	115
rs41247043 rs40724179	CCG CAT TGC CAA GCG GCC TA	TGA CGA AGT CTC CCG CTG GCT	194

1 SNP names from http://www.ncbi.nlm.nih.gov/snp/, SNP ultimately used for genotyping in this study is in bold.

### Generation of rat monoclonal against UV opsin

Generation of rat monoclonal antibodies against UV opsin was performed by Immunoprecise Antibodies Ltd (Victoria BC, Canada) using standard intraperitoneal injection method. Two F344 female rats were immunized with a recombinant antigen designed to mimic the N-terminus of trout UV opsin (NCBI accession NP_001117793.1) [69], [70], see Figure S2E. Sera from hyperimmunized rats had been found to be specific against both trout and zebrafish UV opsin in immunohistochemistry and/or Western blots [16], [34]. Lymphocytes were harvested from the spleen of the best responding rat and fused with rat myeloma YB2/0 to generate the hybridomas. Supernatants from a panel of clonal cells were screened for robust and specific labeling of zebrafish UV cones, and a successful clone was subcloned to generate line 10C9.1.

### Immunocytochemistry and in situ hybridization

Immunocytochemistry was performed on larval zebrafish and retinal sections as previously described [33] to label relevant structures. Briefly, larvae were fixed in 4% paraformaldehyde with 5% sucrose made in PO4, pH 7.4 (PFA) overnight at 4°C. Following fixation, washes of 1.0 M PO_4_/5% sucrose, 1% Tween/H2O (pH 7.4), and acetone were performed. Blocking was done for 90 minutes with 10% NGS/PBS^3+^ (PBS^3+^— phosphate buffered saline with 1% Tween, 1% Triton-X and 1% DMSO, pH 7.4), followed by incubation in antibody in 2% NGS/PBS^3+^ overnight at 4°C. Primary antibodies and dilutions are as follows: 4C12 anti-rod opsin (ZFIN ID: ZDB-ATB-090506-2, 1∶200) [71], zpr-3 anti-rod opsin (ZFIN ID: ZDB-ATB-081002-45, 1∶200); zpr-1 anti-arrestin3a labels double cones (ZFIN ID: ZDB-ATB-081002-43); and 10C9.1 anti-UV opsin (generated herein as described above, 1∶100). Larvae were then incubated in secondary antibody in 2% NGS/PBS^3+^ overnight at 4°C. Secondary antibodies used are as follows: Alexafluor anti-mouse 555 (Invitrogen, Carlsbad, Cat. #A-31570) (1∶1000), Alexafluor anti-rabbit 488 (Invitrogen, Carlsbad, Cat. #A-21441) (1∶1000). Deviations from the above protocol include using PBS/0.01% Tween in lieu of PBS^3+^ and omitting the 1.0 M PO_4_/5% sucrose wash. Retinas were dissected from the head and flatmounted for imaging. Immunohistochemistry on retinal sections followed the same protocols applied instead to 10 μm cryosections of adult eyes prepared as described previously [33] [53].

In situ hybridization for photoreceptor opsins and *tbx2b* expression was performed as previously described [15] using DIG- and FLR-labeled riboprobes against blue-sensitive cone opsin (opn1sw2, 1424 bp, Accession No. AF109372, ZFIN ID: ZDB-GENE-990604-40), UV-sensitive cone opsin (opn1sw1, 1777 bp, Accession No. NM_131319, ZFIN ID: ZDB-GENE-991109-25), rod opsin (1584 bp, Accession No. NM_131084, ZFIN ID: ZDB-GENE-990415-271), or *tbx2b* (1144 bp) [72] (2616 bp, Accession No. NM_131051, ZFIN ID: ZDB-GENE-990726-27). Briefly, larvae were fixed in 4% PFA overnight at 4°C, then permeabilized in MeOH overnight at −20°C. Larvae underwent 30 minutes of digest in Proteinase K at 37°C and were incubated in Hauptmann's prehybridization solution at 65°C for between 2 hours and overnight, depending on the riboprobes used. Subsequently larvae were hybridized with riboprobe for at least one night at 65°C. Blocking with Maleate Tw/2% DMSO/2% RMB was done for 2 hours prior to riboprobe detection using either 1∶100 anti-digoxigenin-POD (Roche, Québec, Cat. # 11 207 733 910) or 1∶100 anti-fluorescein-POD (Roche, Québec, Cat. #11 426 346 910) overnight at 4°C. Larvae were then incubated in tyramide-conjugated fluorochrome according to manufacturer's instructions (Invitrogen, Carlsbad, Cat. #T-20912; T-30954).

Imaging was performed with a Zeiss Axio Observer.Z1 microscope with AxioCam software (Carl Zeiss MicroImaging, Oberkochen). Images were manipulated for channel colour and brightness in AxioCam (Carl Zeiss MicroImaging, Oberkochen), Imaris ×64 (version 7.4.0, Bitplane, Badenerstrasse), or Adobe Photoshop CS5 Extended (Adobe Systems Inc., San Jose).

### Histology

Paraffin sections of adult zebrafish heads fixed in PFA were prepared using standard protocols. Staining of sections with hematoxylin and eosin occurred after dewaxing. Sections were imaged on an Axioscope A.1 microscope (Carl Zeiss MicroImaging, Oberkochen) using a 12-bit, 2 megapixel MacroFIRE colour camera (Optronics, Goleta CA).

### Data analysis

Photoreceptor abundance was measured in flatmounted retinas by counting labeled cells within a 100 μm×100 μm area dorsal to the optic nerve head or, if this location was not obvious, sampling an area containing a minimum of 100 cells of each labeled photoreceptor type. Eye-to-body ratios were calculated using values from measuring the largest width of the eye, and the body length from nose to end of notochord. Cell counts and eye-to-body measurements were performed in ImageJ 1.45 (Wayne Rasband, National Institutes of Health, Bethesda; http://rsbweb.nih.gov/ij/index.html) and statistical analysis was performed in SYSTAT 12 (Systat Software Inc., Chicago) and R (Robert Gentleman and Ross Ihaka, University of Auckland; http://www.r-project.org).

## Supporting Information

Figure S1 **Eye size in various compound mutants shows no obvious change in severity of the microphthalmia phenotype (compare to** **Figure 1C** **).** **A**. Eye diameter along the anterior-posterior axis (orange line) was measured at 6dpf and normalized to body length (not including tail fin) (yellow line). Both normophthalmic and microphthalmic larvae are shown. **B**. Ratios of eye length to body length among the progeny of an in-cross of *tbx2b^+/lor^* fish show no obvious difference from wild type fish, (n = 220). **C**. The same ratios among the progeny of an in-cross of [*gdf6a^+/s327^;tbx2b^+/fby^*] fish show the expected Mendelian abundance of ∼25% microphthlamic fish (see also Fig 3B). The normophthalmic fish have eye sizes distributed in a normal fashion (Shapiro-Wilk Normality test, p>0.05). Among the microphthalmic progeny, there is also a normal distribution of eye size (Shapiro-Wilk test, p>0.05) (n = 194).
(TIF)

Figure S2 **Antibody 10C9.1 specifically labels the outer segments of a class of short single cones in the adult zebrafish retina, as seen in** **Figure 5** **.** **A**. Localization of 10C9.1 labelling to single cone outer segments as clarified by Bodipy counterstain of lipid-rich photoreceptor cell bodies and outer segments. **B–D**. 10C9.1 specificity is supported by localized labeling in the adult retina (B), a lack of labeling when adjacent retinal cryosections are treated identically except for omission of primary antibody (C), and by a dramatic decrease in number of cells labeled when 10C9.1 is applied to retinas from adult zebrafish mutants (*tbx2b^lor/lor^*) that have a paucity of UV cones (D). Other negative controls included applying other rat IgGs as primary antibody, and these produced equivalent results to panel C. Retinas in panels B and D were treated identically including equivalent application of 10C9.1 antibody, and simultaneous processing of tissue by inclusion in the same tissue block prior to cryosectioning. The specificity of 10C9.1 is supported by the paucity of labeling in *tbx2b^lor/lor^* retinas (D), which are known to have few UV cones. Scale bars 30 μm. “rods” indicates rod outer segments; dc, double cones; ipl; inner plexiform layer; onl, outer nuclear layer; inl, inner nuclear layer; rgc, retinal ganglion cell layer. **E**. An alignment of the antigen used to raise 10C9.1 in rats, which represents the 20 N-terminal amino acids from rainbow trout UV opsin plus a C-terminal cysteine to enable linkage of the peptide to the carrier protein keyhole limpet hemocyanin.
(TIF)

Figure S3 **10C9.1 colocalizes with existing rabbit anti-UV antibody (provided by David Hyde, University of Notre Dame).** Scale bar 30 μm. Both the 10C9.1 rat anti-UV and Hyde rabbit anti-UV are somewhat over-exposed to demonstrate background/autofluorescent labeling.
(TIF)

Movie S1 **A new monoclonal antibody raised in rat (10C9.1) labels zebrafish UV cone outer segments.** Coordinate with Figure 5E. Further evidence that rat monoclonal antibody 10C9.1 (pseudocoloured magenta) labels UV cone outer segments. 10C9.1 co-localizes with UV cones filled with green fluorescent protein (GFP, pseudocoloured green), and is excluded from blue cones filled with mCherry (mCh, pseudocoloured cyan) in transgenic zebrafish (*Tg(-5.5opn1sw1:EGFP)kj9;Tg(-3.5opn1sw2:mCherry)ua3011*). Scale bars 30 μm.
(MOV)
