# Supplementary figures and images for: gdf6a Is Required for Cone Photoreceptor Subtype Differentiation and for the Actions of tbx2b in Determining Rod Versus Cone Photoreceptor Fate
Source: PLoS One. 2014 Mar 28;9(3):e92991. doi: 10.1371/journal.pone.0092991 (PMC3969374; doi:10.1371/journal.pone.0092991)

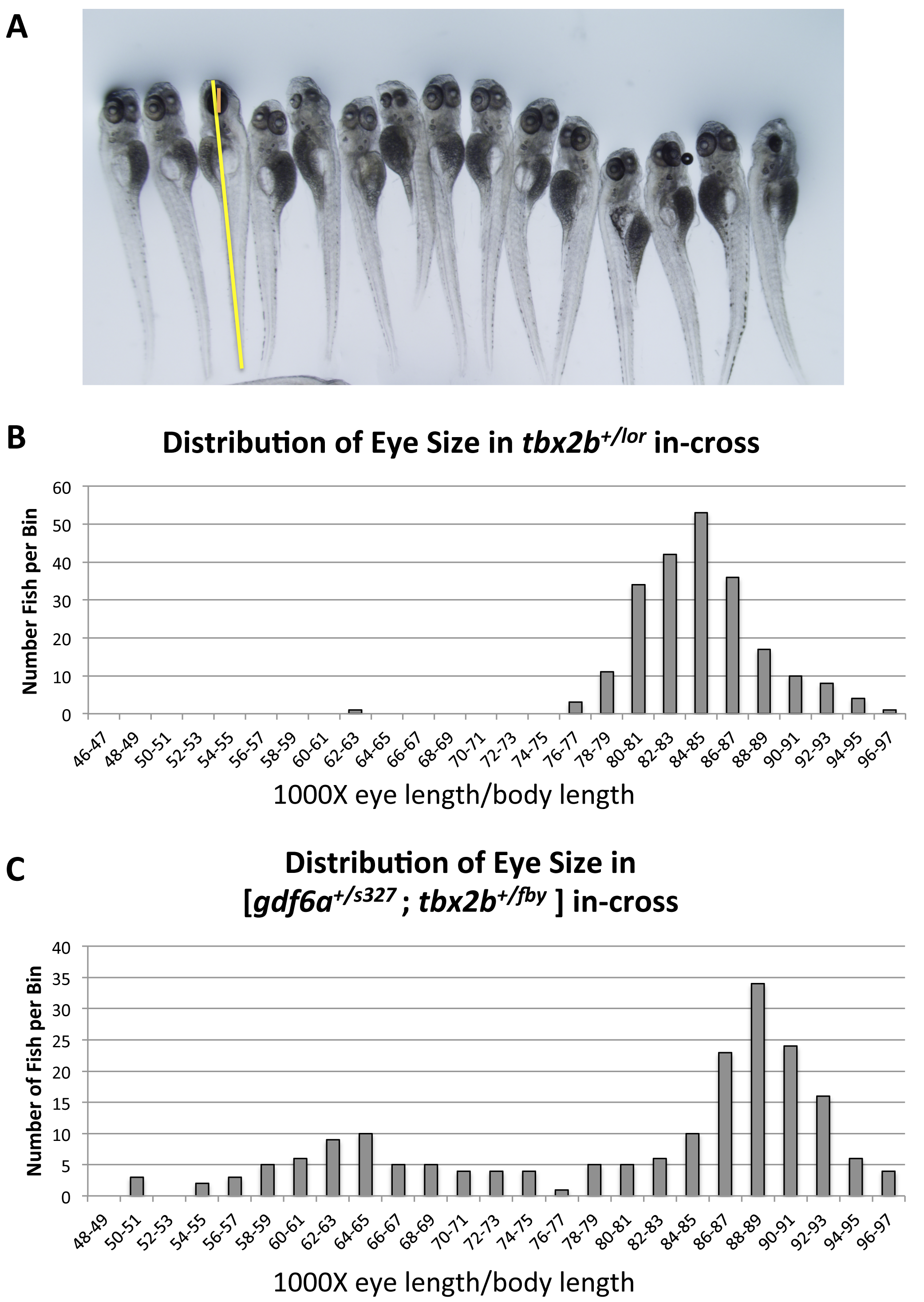

Supplement: Figure S1 — Eye size in various compound mutants shows no obvious change in severity of the microphthalmia phenotype (compare to Figure 1C ). A. Eye diameter along the anterior-posterior axis (orange line) was measured at 6dpf and normalized to body length (not including tail fin) (yellow line). Both normophthalmic and microphthalmic larvae are shown. B. Ratios of eye length to body length among the progeny of an in-cross of tbx2b+/lor fish show no obvious difference from wild type fish, (n = 220). C. The same ratios among the progeny of an in-cross of [gdf6a+/s327;tbx2b+/fby] fish show the expected Mendelian abundance of ∼25% microphthlamic fish (see also Fig 3B). The normophthalmic fish have eye sizes distributed in a normal fashion (Shapiro-Wilk Normality test, p>0.05). Among the microphthalmic progeny, there is also a normal distribution of eye size (Shapiro-Wilk test, p>0.05) (n = 194). (TIF) [file pone.0092991.s001.tif]

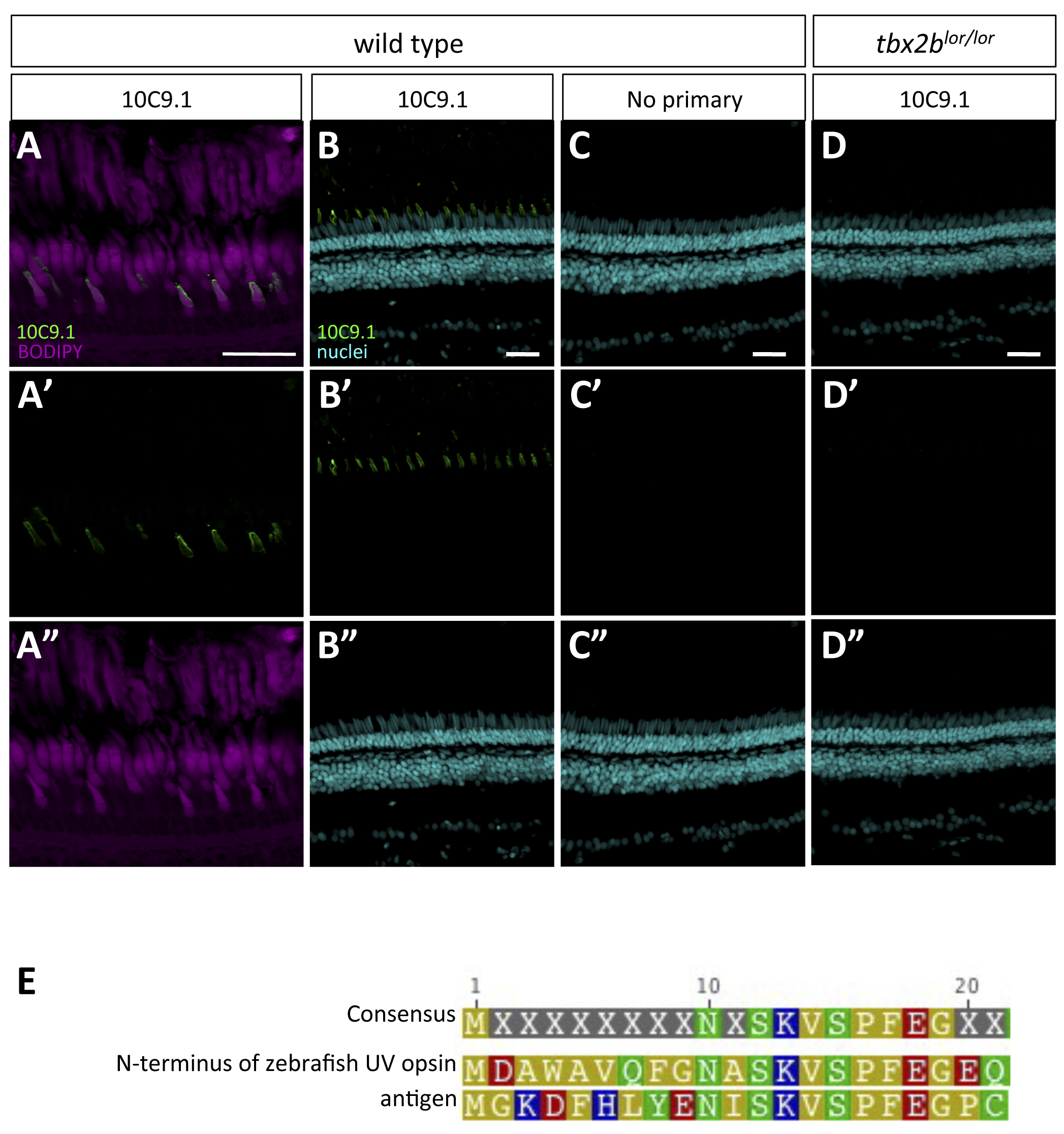

Supplement: Figure S2 — Antibody 10C9.1 specifically labels the outer segments of a class of short single cones in the adult zebrafish retina, as seen in Figure 5 . A. Localization of 10C9.1 labelling to single cone outer segments as clarified by Bodipy counterstain of lipid-rich photoreceptor cell bodies and outer segments. B–D. 10C9.1 specificity is supported by localized labeling in the adult retina (B), a lack of labeling when adjacent retinal cryosections are treated identically except for omission of primary antibody (C), and by a dramatic decrease in number of cells labeled when 10C9.1 is applied to retinas from adult zebrafish mutants (tbx2blor/lor) that have a paucity of UV cones (D). Other negative controls included applying other rat IgGs as primary antibody, and these produced equivalent results to panel C. Retinas in panels B and D were treated identically including equivalent application of 10C9.1 antibody, and simultaneous processing of tissue by inclusion in the same tissue block prior to cryosectioning. The specificity of 10C9.1 is supported by the paucity of labeling in tbx2blor/lor retinas (D), which are known to have few UV cones. Scale bars 30 μm. “rods” indicates rod outer segments; dc, double cones; ipl; inner plexiform layer; onl, outer nuclear layer; inl, inner nuclear layer; rgc, retinal ganglion cell layer. E. An alignment of the antigen used to raise 10C9.1 in rats, which represents the 20 N-terminal amino acids from rainbow trout UV opsin plus a C-terminal cysteine to enable linkage of the peptide to the carrier protein keyhole limpet hemocyanin. (TIF) [file pone.0092991.s002.tif]

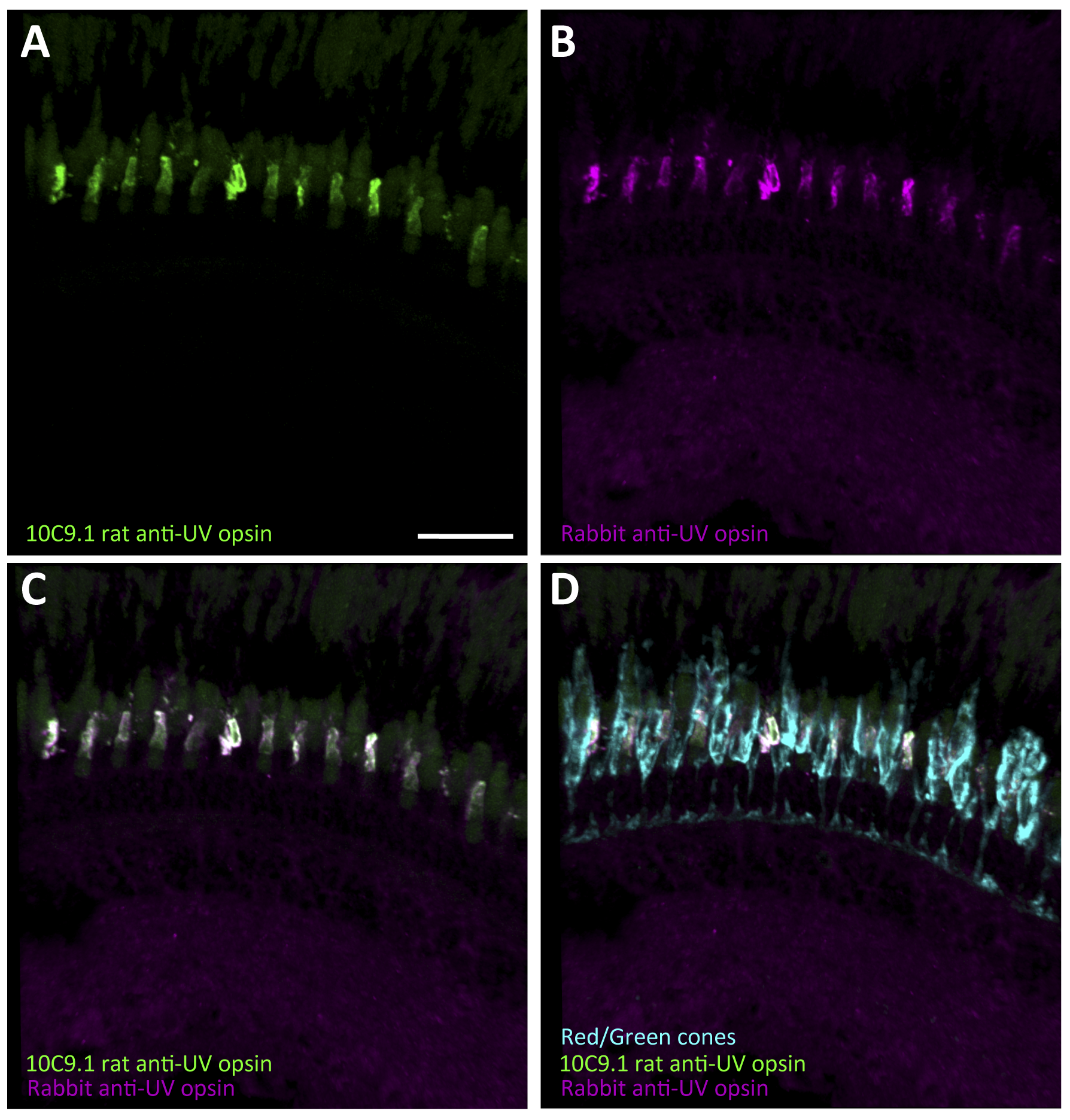

Supplement: Figure S3 — 10C9.1 colocalizes with existing rabbit anti-UV antibody (provided by David Hyde, University of Notre Dame). Scale bar 30 μm. Both the 10C9.1 rat anti-UV and Hyde rabbit anti-UV are somewhat over-exposed to demonstrate background/autofluorescent labeling. (TIF) [file pone.0092991.s003.tif]
